# The histone deacetylases Rpd3 and Hst1 antagonistically regulate *de novo* NAD^+^ metabolism in the budding yeast *Saccharomyces cerevisiae*

**DOI:** 10.1016/j.jbc.2022.102410

**Published:** 2022-08-22

**Authors:** Benjamin Groth, Chi-Chun Huang, Su-Ju Lin

**Affiliations:** Department of Microbiology and Molecular Genetics, College of Biological Sciences, University of California, Davis, California, USA

**Keywords:** NAD^+^ biosynthesis, cell metabolism, metabolic regulation, yeast genetics, yeast metabolism, histone deacetylase, gene regulation, *BNA*, biosynthesis of nicotinic acid, ChIP, chromatin immunoprecipitation, 3-HA, 3-hydroxyanthranilic acid, HDAC, histone deacetylase, 3-HK, 3-hydroxykynurenine, KYN, kynurenine, MS, mass spectrometry, NA, nicotinic acid, NAM, nicotinamide, NaMN, nicotinic acid mononucleotide, NMN, nicotinamide mononucleotide, NR, nicotinamide riboside, QA, quinolinic acid, qRT, quantitative RT, SC, synthetic complete, SD, synthetic defined, TRP, l-tryptophan

## Abstract

NAD^+^ is a cellular redox cofactor involved in many essential processes. The regulation of NAD^+^ metabolism and the signaling networks reciprocally interacting with NAD^+^-producing metabolic pathways are not yet fully understood. The NAD^+^-dependent histone deacetylase (HDAC) Hst1 has been shown to inhibit *de novo* NAD^+^ synthesis by repressing biosynthesis of nicotinic acid (*BNA*) gene expression. Here, we alternatively identify HDAC Rpd3 as a positive regulator of *de novo* NAD^+^ metabolism in the budding yeast *Saccharomyces cerevisiae*. We reveal that deletion of *RPD3* causes marked decreases in the production of *de novo* pathway metabolites, in direct contrast to deletion of *HST1*. We determined the *BNA* expression profiles of *rpd3*Δ and *hst1Δ* cells to be similarly opposed, suggesting the two HDACs may regulate the *BNA* genes in an antagonistic fashion. Our chromatin immunoprecipitation analysis revealed that Rpd3 and Hst1 mutually influence each other’s binding distribution at the *BNA2* promoter. We demonstrate Hst1 to be the main deacetylase active at the *BNA2* promoter, with *hst1Δ* cells displaying increased acetylation of the N-terminal tail lysine residues of histone H4, H4K5, and H4K12. Conversely, we show that deletion of *RPD3* reduces the acetylation of these residues in an Hst1-dependent manner. This suggests that Rpd3 may function to oppose spreading of Hst1-dependent heterochromatin and represents a unique form of antagonism between HDACs in regulating gene expression. Moreover, we found that Rpd3 and Hst1 also coregulate additional targets involved in other branches of NAD^+^ metabolism. These findings help elucidate the complex interconnections involved in effecting the regulation of NAD^+^ metabolism.

NAD^+^ is a metabolite with crucial roles in a variety of cellular processes. It is involved in the oxidative steps of glycolysis and in mitochondrial energy production as a redox cofactor, in epigenetic regulation as a cosubstrate for class III histone deacetylases (HDACs), also known as sirtuins (1, 2, 3), and in DNA repair as a substrate for poly-ADP-ribose polymerases (4). As such, defects of NAD^+^ metabolism are associated with a broad range of disorders, such as diabetes, Alzheimer's disease, and various cancers (5, 6, 7, 8, 9, 10, 11, 12, 13, 14, 15, 16, 17, 18, 19, 20, 21, 22, 23).

NAD^+^ may be produced by three major pathways: (1) *de novo* biosynthesis from l-tryptophan (TRP), (2) salvage of nicotinic acid (NA) and nicotinamide (NAM), and (3) salvage of nicotinamide riboside (NR) (Fig. 1*A*). These pathways are largely conserved, with a few species-specific differences, and consume cellular pools of ATP, phosphoribosyl pyrophosphate, and glutamine (24, 25). The *de novo* pathway is also known as the kynurenine (KYN) pathway of TRP degradation. In yeast, this pathway is characterized by the synthesis of quinolinic acid (QA) from TRP by five enzymatic reactions mediated by the Bna (biosynthesis of nicotinic acid) proteins (Bna2, Bna7, Bna4, Bna5, and Bna1) and a spontaneous cyclization (26). Bna6 then converts QA into nicotinic acid mononucleotide (NaMN), which is also produced by the NA–NAM salvage branch (Fig. 1*A*). Under standard NA-abundant growth conditions, NA–NAM salvage is the preferred NAD^+^ biosynthesis route (27). NAM may come from NAD^+^-consuming reactions including sirtuin-mediated protein deacetylation (1, 2, 3). NAM can be converted to NA by a nicotinamidase Pnc1 (28), leading to NaMN production by Npt1 (Fig. 1*A*). Although human cells do not have Pnc1-like nicotinamidases, recent studies suggest that NAM salvage to NA may also take place in humans with the aid of gut bacterial nicotinamidases (29). In the NR salvage branch, NR can enter the NA–NAM salvage branch when converted to NAM by nucleotidases Urh1 and Pnp1 (30, 31). NR can also be converted to nicotinamide mononucleotide (NMN) by the NR kinase, Nrk1 (32). NMN adenylyltransferases (Nma1, Nma2, and Pof1 in yeast) are responsible for the conversion of NMN to NAD^+^ (33, 34, 35). Nma1 and Nma2 also convert NaMN to nicotinic acid adenine dinucleotide (33, 34), which is converted to NAD^+^ by the glutamine-dependent NAD^+^ synthetase Qns1 (36). Under NAD^+^-repleted conditions, the *de novo* pathway *BNA* genes are repressed by the NAD^+^-dependent sirtuin Hst1 (37, 38). Conversely, NAD^+^ depletion results in decreased Hst1 activity leading to transcription activation of the *BNA* genes. Yeast cells also release and reuptake small NAD^+^ precursors, such as NA, NAM, QA, and NR (Fig. 1*A*) (35, 38, 39, 40, 41). The mechanisms of precursor release remain unclear, and it is suggested that vesicular trafficking may play a role (24, 39, 42). Transport of NAD^+^ precursors into yeast cells is mediated by specific transporters Tna1 (for NA and QA) (40, 43) and Nrt1 (for NR) (44).

**Figure 1:**
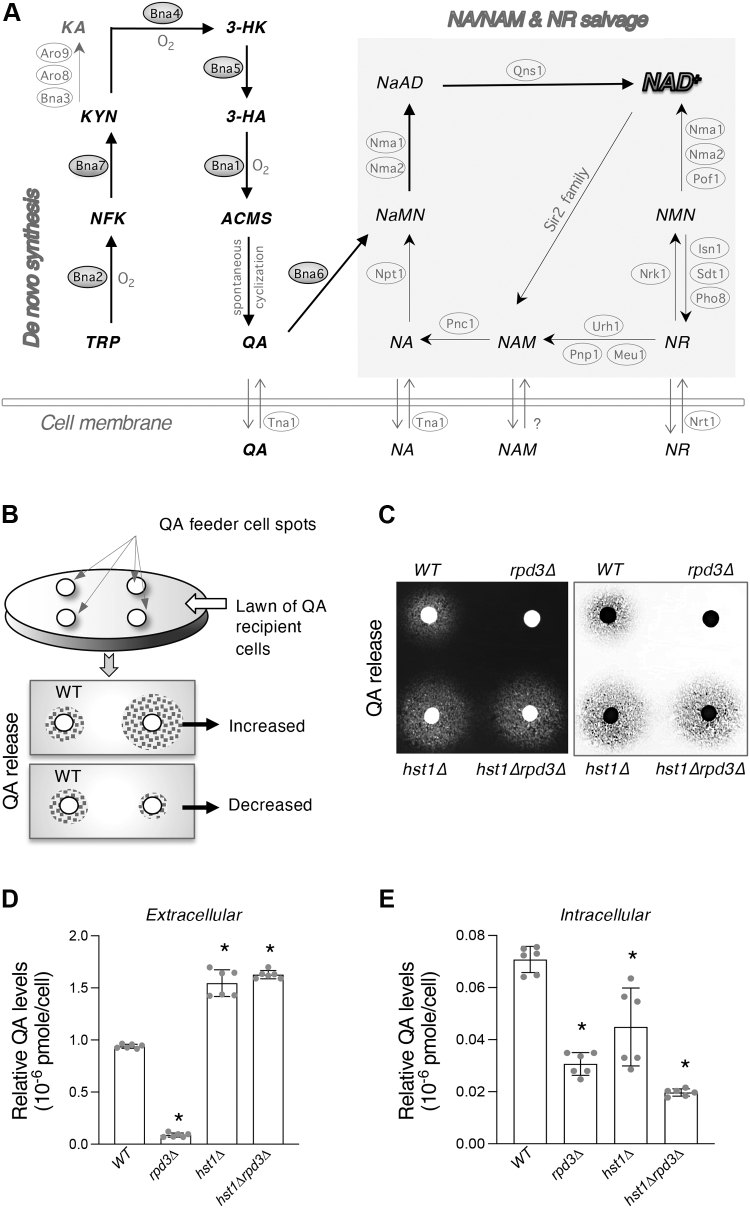
**Cells lacking *RPD3* are deficient for *de novo* QA production.***A*, model of the NAD^+^ biosynthetic pathways in *Saccharomyces cerevisiae*. *De novo* NAD^+^ metabolism begins with TRP, which is converted into NaMN by the Bna enzymes (Bna2, Bna7, Bna4, Bna5, Bna1, and Bna6) (*left*). NaMN is also produced by salvage of NA and NAM, which is further connected with salvage of NR (*right*). NR is metabolized to NMN by Nrk1, which is then converted to NAD^+^ by Nma1, Nma2, and Pof1. Abbreviations of NAD^+^ intermediates are shown in *bold* and *italicized*. 3-HA, 3-hydroxyanthranilic acid; 3-HK, 3-hydroxykynurenine; ACMS, 2-amino-3-carboximuconate-6-semialdehyde; KA, kynurenic acid; KYN, kynurenine; NA, nicotinic acid; NaAD, deamido-NAD^+^; NAM, nicotinamide; NaMN, nicotinic acid mononucleotide; NFK, *N*-formylkynurenine; NMN, nicotinamide mononucleotide; NR, nicotinamide riboside; QA, quinolinic acid; TRP, l-tryptophan. Abbreviations of protein names are shown in *ovals*. Aro9/Aro8 and Bna3, kynurenine aminotransferase; Bna1, 3-hydroxyanthranilate 3,4-dioxygenase; Bna2, tryptophan 2,3-dioxygenase; Bna4, kynurenine 3-monooxygenase; Bna5, kynureninase; Bna6, quinolinic acid phosphoribosyl transferase; Bna7, kynurenine formamidase; Nma1/2, NaMN/NMN adenylyltransferase (NMNAT); Npt1, nicotinic acid phosphoribosyl transferase; Pof1, NMN adenylyltransferase (NMNAT); Pnc1, nicotinamidase; Qns1, glutamine-dependent NAD^+^ synthetase; Sir2 family, NAD^+^-dependent protein deacetylases; Urh1, Pnp1, and Meu1, nucleosidases; Nrk1, NR kinase; Isn1 and Sdt1, nucleotidases; Pho8 and Pho5, phosphatases. Tna1, NA, and QA transporter; Nrt1, NR transporter. *B*, illustration of the QA cross-feeding assay used to determine relative levels of QA release in strains of interest. Spots of haploid single-deletion feeder cells were applied to a lawn of QA-dependent recipient cells (*bna4Δnrk1Δnpt1Δ*) and allowed to grow for 2 to 3 days at 30 °C. The density of recipient cell growth around the feeder cell spots correlates with the amount of QA released by the feeder cells. *C*, deletion of *RPD3* (*rpd3Δ*) decreases QA cross-feeding activities, whereas deletion of *HST1* (*hst1Δ*) as well as *HST1* and *RPD3* together (*hst1Δrpd3Δ*) increases QA cross-feeding activities. Feeder cell spots along with recipient cells were grown on SC plates at 30 °C for 2 days. For clarity, inverse image is also shown (*right*). *D*, extracellular QA levels determined in the growth media. Deletion of *RPD3* decreases QA release, whereas deletion of *HST1* as well as *HST1* and *RPD3* together increases QA release. *E*, intracellular QA levels determined in the cell lysates. Deletion of *RPD3* decreases intracellular stores of QA. For *D* and *E*, the graphs are based on data of two independent experiments. Error bars represent data from two biological replicates per strain each with three technical replicates (total *n* = 6 per strain). The *p* values are calculated using Student’s *t* test (∗*p* < 0.05). SC, synthetic complete.

Supplementation of precursors in each pathway has been shown to produce positive outcomes in some disease models (6, 20). However, given the complexity of NAD^+^ metabolism and its interaction with other signaling networks, it is often necessary to understand the pathways affected in each particular disease state in order to derive the greatest advantage from treatment and determine the most useful precursor for each case. This represents one major benefit that may be won by the detailed study of the molecular mechanisms governing the regulation of each level of NAD^+^ metabolism. In addition to being crucial for the maintenance of cellular NAD^+^ pools, the intermediates of these pathways often have multifarious other roles (12, 42, 45). For instance, intermediates of *de novo* metabolism have been associated with both beneficial and adverse influences on neurological health (16, 46, 47) and have also been shown to interact with immune signaling (16, 46, 47, 48, 49). Indeed, *de novo* metabolism, despite being a relatively minor contributor to the NAD^+^ pool compared with salvage of NA, NAM, and NR, is increasingly recognized as an important element of NAD^+^ metabolism with a variety of far-ranging influences on cellular health (47, 50).

The regulation of the *de novo* pathway of NAD^+^ biosynthesis is still incompletely understood. Previous studies in yeast have shown the sirtuin Hst1 (37) and the copper-sensing transcription factor Mac1 (38) to be negative regulators of *de novo* metabolism, whereas a complex of Bas1 and Pho2 transcriptionally activates *de novo* metabolism under conditions of adenine depletion (25). In addition, a fragment of the Huntingtin protein was shown to activate production of several *de novo* intermediates, an effect that is ameliorated by the inhibition of class I HDAC Rpd3 (51). Deletion of *RPD3* has also been shown to reduce expression of *BNA1* (52). In this study, we identified Rpd3 as a positive regulator of the majority of the *BNA* genes of *de novo* NAD^+^ biosynthesis, the deletion of which results in markedly diminished production of several *de novo* pathway metabolites. In addition, we characterized the interaction of Rpd3 with the NAD^+^-dependent Hst1 in the regulation of *de novo* NAD^+^ metabolism. This work helps to elaborate the mechanism by which *BNA* expression is regulated and to clarify the connection of *de novo* NAD^+^ metabolism to other branches of metabolism and signaling in the cell.

## Results

### The HDAC Rpd3 is a positive regulator of QA production

The signaling pathways that regulate NAD^+^ metabolism remain unclear in part because of the dynamic nature and complexity of NAD^+^ synthesis pathways. Making use of the tendency of yeast cells to constantly release and retrieve small NAD^+^ precursors (35, 39, 40, 41), we developed genetic screens to identify and study novel NAD^+^ homeostasis factors. The *rpd3Δ* mutant was identified in a screen for mutants that showed altered QA release using a “cross-feeding” assay as described previously (38). As illustrated in Figure 1, *B*, a lawn of QA-dependent “recipient cells” (*bna4Δnpt1Δnrk1Δ*) is first spread onto an agar plate. These cells cannot grow because they are dependent on exogenous QA for NAD^+^ synthesis, whereas standard growth medium does not contain QA. Next, “feeder cells” (strains of interest) are spotted onto the lawn of “recipient cells.” In this manner, the “feeder cells” release QA to support the growth of “recipient cells” by crossfeeding. This assay determines relative levels of total QA produced and released by “feeder cells” and is considered as a readout for *de novo* pathway activity (Fig. 1*B*). The *rpd3Δ* mutant caught our attention because it did not appear to release QA when compared with WT cells (Fig. 1*C*). This is to our surprise, because we previously reported that cells lacking another HDAC, Hst1, exhibited the opposite phenotype (38) (Fig. 1*C*). Interestingly, the *hst1Δrpd3Δ* double mutant showed increased QA release, closely matching those seen for the *hst1Δ* single mutant (Fig. 1*C*), suggesting Hst1 may function downstream of Rpd3. Next, we examined whether the QA cross-feeding deficiency observed in the *rpd3Δ* mutant (Fig. 1*C*) was due to decreased QA production or altered QA transport. To answer this question, we determined the QA content in the cell lysate and growth medium using quantitative liquid assays. Our results showed that the *rpd3Δ* mutant is likely defective in QA production, because it has a significant reduction in both extracellular (Fig. 1*D*) and intracellular QA pools (Fig. 1*E*). Interestingly, despite releasing a significantly higher amount of QA (Fig. 1, *C* and *D*), the *hst1Δ* and *hst1Δrpd3Δ* mutants appeared to have slightly lower intracellular QA levels (Fig. 1*E*). This is in line with our previous report that yeast cells seem to maintain low intracellular QA levels and that excess QA is either released extracellularly or converted to NAD^+^ (38). QA accumulation may be detrimental to cells; for example, it has been linked with the production of reactive oxygen species (16). However, the factors leading *hst1Δ* and *hst1Δrpd3Δ* cells to store less QA remain unclear.

### Cells lacking Rpd3 also show altered NA–NAM salvage, NR salvage, and *de novo* activities

**Figure 2:**
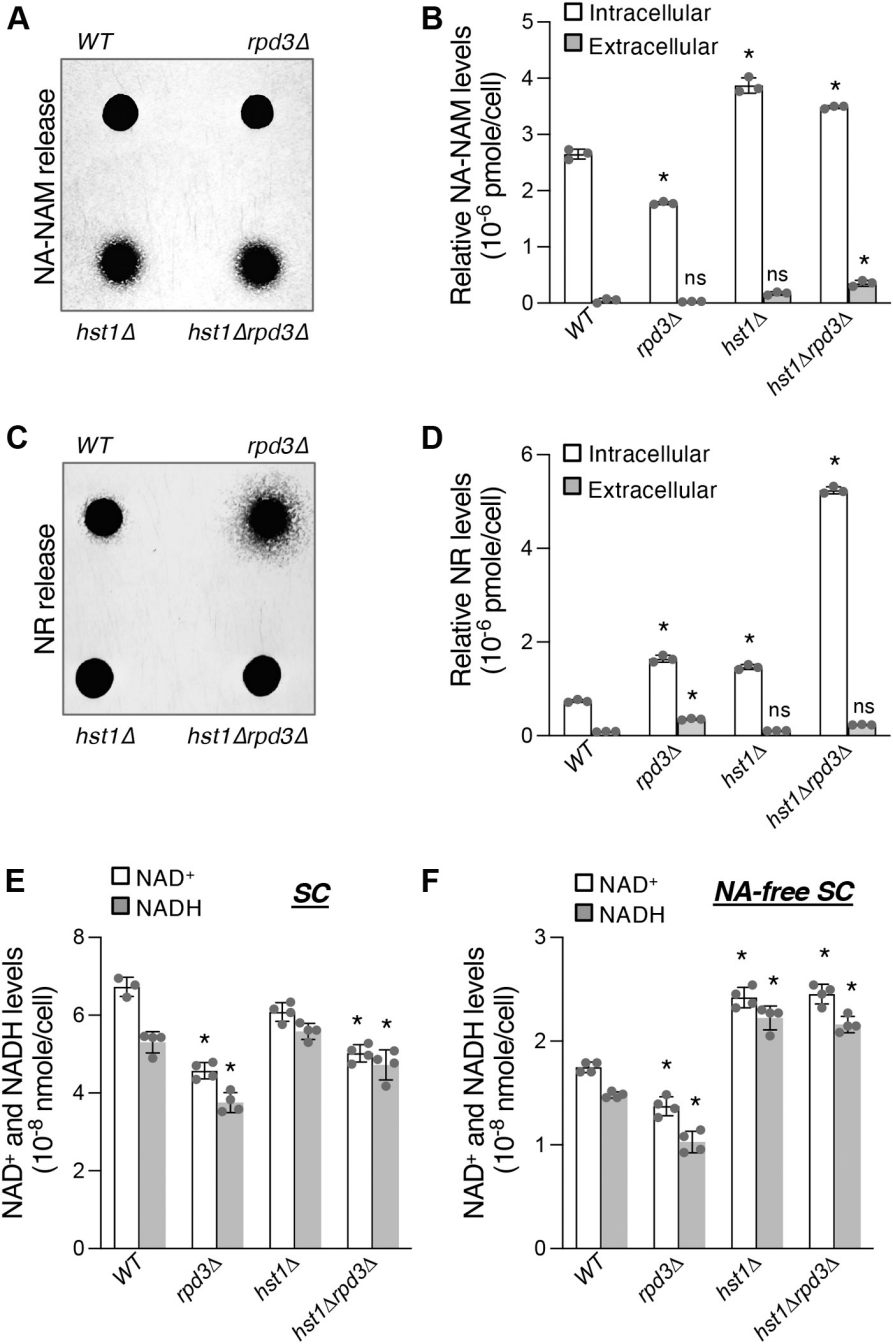
**Determination of NAD**^**+**^**salvage pathway intermediates and NAD**^**+**^**levels in cells lacking *RPD3* and HST1.***A*, *hst1Δ* and *hst1Δrpd3Δ* cells show increased NA–NAM cross-feeding activities. Feeder cells spots along with NA–NAM-dependent recipient cells (*bna6Δnkr1Δnrt1Δ*) were grown on NA-free SC plate at 30 °C for 3 days. *B*, quantification of NA–NAM production by measuring the extracellular (released) and intracellular (stored) levels of NA–NAM. *rpd3Δ* cells show decreased release and intracellular storage of NA–NAM, whereas *hst1Δ* and *hst1Δrpd3Δ* cells show increased release and storage of NA–NAM. *C*, *rpd3Δ* cells show increased NR cross-feeding activities, whereas *hst1Δ* and *hst1Δrpd3Δ* cells show decreased activities. Feeder cell spots along with NR-dependent recipient cells (*npt1Δbna6Δpho5Δ*) were grown at 30 °C on YPD plate for 3 days. *D*, *rpd3Δ* and *hst1Δ* cells show increased intracellular storage of NR. The *hst1Δrpd3Δ* double mutant shows a further increase compared with the single mutants. Only the *rpd3Δ* mutant shows a significant increase in NR release. *E*, *rpd3Δ* and *hst1Δrpd3Δ* cells exhibit significantly reduced NAD^+^ levels in standard SC medium. *F*, *rpd3Δ* cells display reduced NAD^+^ levels in NA-free SC medium, whereas *hst1Δ* and *hst1Δrpd3Δ* display increased NAD^+^ levels. For *B* and *D–F*, graphs are representative of the trend observed across three independent experiments. For *B* and *D*, error bars represent data from three technical replicates for each strain in an experiment. For *E* and *F*, error bars represent data from two biological replicates, each with two technical replicates for each strain in an experiment. The *p* values are calculated using Student’s *t* test (∗*p* < 0.05; *ns*, not significant). NA, nicotinic acid; NAM, nicotinamide; NR, nicotinamide riboside; SC, synthetic complete; YPD, yeast extract/peptone/dextrose.

Next, we examined whether Rpd3 also plays a role in regulating the other two branches of NAD^+^ metabolism, and whether there is an interaction between Rpd3 and Hst1 for this regulation as observed in the regulation of *de novo* QA production. As shown in Figure 2, *A*, the *hst1Δ* and *hst1Δrpd3Δ* mutants showed increased NA–NAM release, whereas the *rpd3Δ* single mutant did not. This suggests that Rpd3 plays a role in NA–NAM salvage and that Hst1 may function downstream of Rpd3. Note that WT cells did not show visible NA–NAM release (Fig. 2*A*), and therefore, it was unclear whether the *rpd3Δ* mutant has reduced or unchanged NA–NAM production. This observation is consistent with a previous report that the majority of NA–NAM is stored intracellularly (39). To address this question, we determined intracellular NA–NAM levels and were able to observe a reduction in NA–NAM levels in *rpd3Δ* cells (Fig. 2*B*). Conversely, release of NR is increased in *rpd3Δ* cells in comparison with WT, whereas little difference is evident in both *hst1Δ* and *hst1Δrpd3Δ* cells (Fig. 2*C*). Interestingly, intracellular NR levels were raised not only in *rpd3Δ* but also in *hst1Δ* cells and, to a remarkably high degree, *hst1Δrpd3Δ* cells (Fig. 2*D*). Although both the *hst1Δ* and *rpd3Δ* mutants showed increased NR production (Fig. 2*D*), increased NR release was only observed in the *rpd3Δ* but not in the *hst1Δ* mutants (Fig. 2*C*). This is likely because of an increase in NR import in the *hst1Δ* mutants since the expression of NR transporter, Nrt1, is increased in these cells (38). These results also suggest some degree of synergy between Rdp3 and Hst1 in the regulation of the NR salvage pathway.

We next investigated the influence of *rpd3Δ* and *hst1Δ* on cellular NAD^+^ pools. As shown in Figure 2, *E*, a moderate, yet significant, reduction in NAD^+^ levels was observed in both *rpd3Δ* and *hst1Δrpd3Δ* mutants (Fig. 2*E*) when cells were grown in standard synthetic complete (SC) media. It has been suggested that NA–NAM salvage is the favored route for NAD^+^ synthesis when NA is present (27) (which is the case for all standard growth media). Therefore, observed NAD^+^ defects in *rpd3Δ* cells may be due to deficient NA–NAM salvage (37, 38). To directly examine whether the *rpd3Δ* mutant is defective in *de novo* NAD^+^ synthesis, cells were cultivated in medium lacking NA (NA-free SC), removing exogenous precursors for the salvage pathways and isolating *de novo* metabolism for analysis. As shown in Figure 2, *F*, decreased NAD^+^ levels were observed in *rpd3Δ* cells (Fig. 2*F*), which was in line with the defects in QA production exhibited by these cells (Fig. 1, *C* and *D*). Conversely, NAD^+^ levels were raised to a similar degree in both *hst1Δ* and *hst1Δrpd3Δ* cells, in alignment with the increased QA production observed in both strains (Fig. 1*D*). This also suggests that the reduced levels of intracellular QA observed in these two strains (Fig. 1*E*) may be due to more efficient NAD^+^ synthesis.

**Figure 3:**
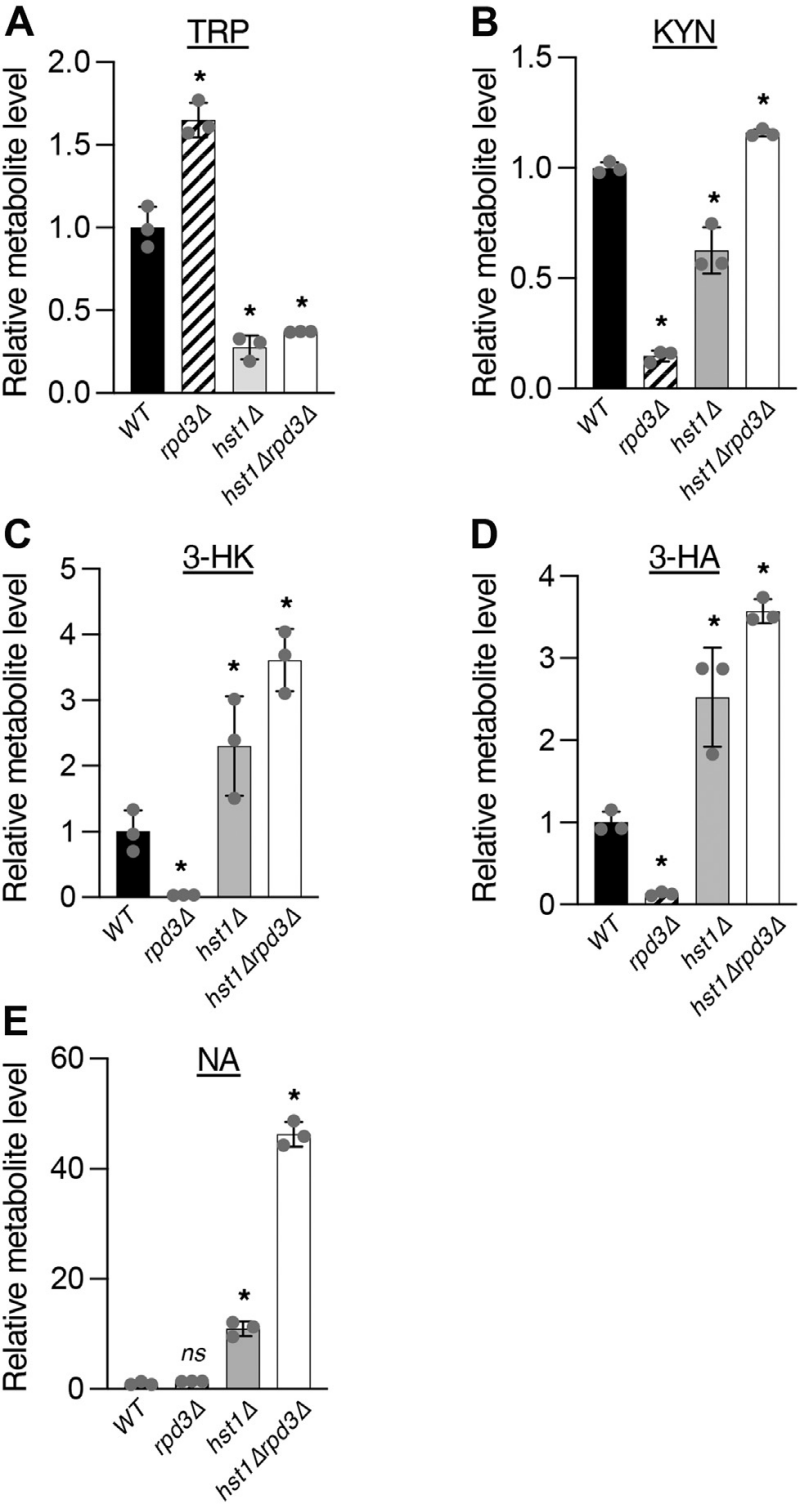
**Rpd3 and Hst1 regulate homeostasis of *de novo* intermediates.***A*, mass spectrometry analysis of TRP levels in *rpd3Δ*, *hst1Δ*, and *hst1Δrpd3Δ* cells. Deletion of *RPD3* leads to accumulation of TRP, whereas *hst1Δ* and *hst1Δrpd3Δ* cells show reduced TRP levels. *B*, *rpd3Δ* cells exhibit defective KYN production. *C*, *rpd3Δ* cells show reduced 3-HK levels, whereas *hst1Δ* and *hst1Δrpd3Δ* cells show increased 3-HK levels. *D*, *rpd3Δ* cells produce reduced levels of 3-HA, whereas *hst1Δ* and *hst1Δrpd3Δ* cells produce greater levels of 3-HA. *E*, deletion of *HST1* and especially deletions of *RPD3* and *HST1* together increase NA levels. All values for each metabolite are normalized to levels in WT cells. Error bars represent data from three technical replicates. The *p* values are calculated using Student’s *t* test (∗*p* < 0.05; *ns*, not significant). 3-HA, 3-hydroxyanthranilic acid; 3-HK, 3-hydroxykynurenine; KYN, kynurenine; NA, nicotinic acid; TRP, l-tryptophan.

In order to determine whether the defects seen in QA and NAD^+^ production were reflective of a general dysfunction in *de novo* NAD^+^ metabolism (Fig. 1*A*), we analyzed several intermediates of the pathway present in the cell lysate by mass spectrometry (MS). In accordance with expectations, *rpd3Δ* cells displayed increased TRP levels, whereas *hst1Δ* and *hst1Δrpd3Δ* cells showed reduced accumulation of TRP (Fig. 3*A*). This suggests that *rpd3Δ* cells are deficient for TRP utilization *via* the *de novo* NAD^+^ biosynthesis pathway, whereas *hst1Δ* and *hst1Δrpd3Δ* cells consume more TRP because of increased *de novo* pathway activity. Indeed, *rpd3Δ* cells showed reduced levels of KYN (Fig. 3*B*), 3-hydroxykynurenine (3-HK) (Fig. 3*C*), and 3-hydroxyanthranilic acid (3-HA) (Fig. 3*D*). Although *hst1Δ* cells showed reduced levels of KYN, an early intermediate of *de novo* NAD^+^ metabolism, both *hst1Δ* and *hst1Δrpd3Δ* cells exhibited increased levels of the downstream intermediates 3-HK and 3-HA, likely because of the rapid flux of *de novo* metabolism expected for cells lacking Hst1 (38). Because of the extremely low concentration of QA stored in the cell (Fig. 1*E*) (38), we were unable to detect QA in the cell lysate by MS using same standard conditions. Since most excess QA is released extracellularly, the QA cross-feeding plate assay appears to be a convenient readout for released QA. Moreover, the plate assay measures QA accumulation in the growth media during the entire time course of recipient cell growth, and therefore, the readout signal may be amplified. MS analysis also confirmed increased intracellular NA accumulation in *hst1Δ* and *hst1Δrpd3Δ* cells (Fig. 3*E*), suggested by the cross-feeding studies of NA–NAM (Fig. 2, *A* and *B*).

### Rpd3 is important for optimal activation of the *BNA* genes in *de novo* metabolism

**Figure 4:**
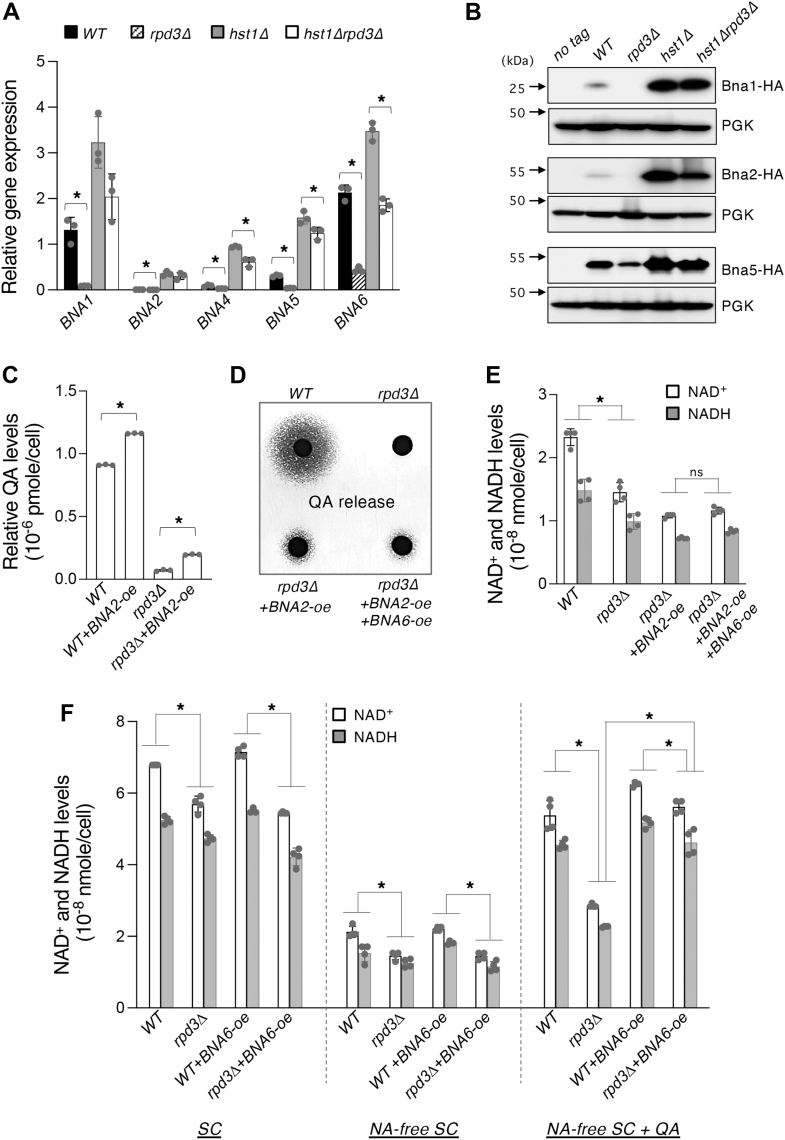
**Rpd3 positively regulates *de novo* NAD**^**+**^**metabolism.***A*, gene expression quantitative PCR (qPCR) analysis of *BNA* mRNA in WT, *rpd3Δ*, *hst1Δ*, and *hst1Δrpd3Δ* cells. Values shown are relative expression levels normalized to *TAF10* as a control. Deletion of *RPD3* decreases expression of all *BNA* genes shown. *BNA* expression in *hst1Δrpd3Δ* cells is generally increased relative to WT cells and slightly less than levels in *hst1Δ* cells. *B*, comparisons of Bna protein expression in HDAC mutants. HA-tagged Bna1, Bna2, and Bna5 proteins were generated in WT, *rpd3Δ*, *hst1Δ*, and *hst1Δrpd3Δ* cells. Protein expression was determined by Western blot analysis. *Arrows* make the positions of molecular weight markers. *C*, overexpression of *BNA2* (*BNA2-oe*) slightly increases the levels of QA release. *D*, *BNA2-oe* increases QA release in *rpd3Δ*, whereas overexpression of both *BNA2* and *BNA6* in *rpd3Δ* clears accumulated QA. *E*, *BNA2-oe* alone or *BNA2-oe* and *BNA6-oe* together is insufficient to raise NAD^+^ levels in *rpd3Δ* cells grown in SC. *F*, restoration of *de novo* pathway activity is necessary to rescue NAD^+^ levels in *rpd3Δ* cells. NAD^+^ levels in *rpd3Δ* cells are increased to WT levels when supplemented with QA (at 10 μM) and with *BNA6-oe*. For *A*, *C*, *E*, and *F*, the graphs are representative of the trend observed across three independent experiments. For *A* and *C*, error bars represent data from three technical replicates for each strain in an experiment. For *E* and *F*, error bars represent data from two biological replicates each with two technical replicates for each strain in an experiment. The *p* values are calculated using Student’s *t* test (∗*p* < 0.05; *ns*, not significant). BNA, biosynthesis of nicotinic acid; HDAC, histone deacetylase; QA, quinolinic acid.

To further study the role of Rpd3 in NAD^+^ metabolism, we first asked whether the defects in *de novo* NAD^+^ metabolism shown in *rpd3Δ* cells were due to dysregulation of the *BNA* gene expression (Fig. 1*A*). As expected, *rpd3Δ* cells showed strongly reduced expression of most of the *BNA* genes involved in *de novo* metabolism (Fig. 4*A*). *BNA7* appears to be insensitive to the factors affecting the other *BNA* genes (38), whereas *BNA3* is not strictly a part of the course of the *de novo* pathway (Fig. 1*A*) (53); hence, the two were not investigated. Consistent with previous studies (37, 38), *hst1Δ* cells exhibited markedly increased *BNA* expression, whereas *hst1Δrpd3Δ* cells showed an expression phenotype most closely resembling *hst1Δ*, yet in most cases slightly but significantly below the levels seen in *hst1Δ* alone (Fig. 4*A*). The ability of *hst1Δrpd3Δ* to override the expression deficits observed in *rpd3Δ* cells suggests that Hst1 functions downstream of Rpd3 and that Rpd3 promotes transcription possibly by opposing the repressive activity of Hst1 on the *BNA* gene promoters. However, additional factors are likely involved, as *BNA* gene expression in *hst1Δrpd3Δ* cells was not fully restored to level observed in *hst1Δ* cells (Fig. 4*A*). We further confirmed that observed *BNA* expression changes were reflective of Bna protein expression. As shown in Figure 4, *B*, significant decreases of the three Bna proteins examined (Bna1, Bna2, and Bna5) were observed in *rpd3Δ* cells, whereas significant increases of these proteins were observed in *hst1Δ* and *hst1Δrpd3Δ* cells.

Having shown Rpd3 to be a positive regulator of *BNA* expression, we then sought to investigate whether decreased *BNA* expression is ultimately responsible for the low QA and NAD^+^ levels in *rpd3Δ* cells. We began with *BNA2*, the rate-limiting enzyme of the *de novo* pathway (38), and inquired whether restoration of this step could replenish the low QA levels seen in *rpd3Δ* cells. We found that overexpression of *BNA2* (from the *ADH1* promoter, so as to decouple it from regulation by Rpd3) was only sufficient to induce a small but significant increase in QA levels, which nevertheless remained far below those seen in WT cells (Fig. 4, *C* and *D*). Pairing overexpression of *BNA2* with *BNA6*, which increases QA assimilation into NAD^+^ (Fig. 1*A*), we found that *BNA6*-oe effectively cleared the small amount of QA accumulated in *rpd3Δ BNA2*-oe cells (Fig. 4*D*). We then examined whether this modest increase of QA induced by *BNA2*-oe could restore the NAD^+^ levels of *rpd3Δ* cells. As shown in Figure 4, *E*, neither *BNA2*-oe alone nor *BNA2*-oe + *BNA6*-oe was sufficient to stimulate any visible increase in the NAD^+^ pool in *rpd3Δ* cells. This indicated that each step of *de novo* metabolism would need to be accounted for in order to achieve full restoration of NAD^+^ levels in *rpd3Δ* cells. To test whether this was the case, we attempted to reinstate *de novo* activity by bypassing the earlier steps of the pathway. To achieve this, we cultured cells in NA-free SC supplemented with QA and overexpressed *BNA6* (Fig. 4*F*, *right*), feeding directly into the main pathway of NAD^+^ biosynthesis *via* NaMN (Fig. 1*A*). As controls, we also included cells grown in standard SC (Fig. 4*F*, *left*) and NA-free SC (Fig. 4*F*, *middle*) without QA supplementation. As shown in Figure 4, *F* (*right*), neither QA supplementation nor *BNA6*-oe alone was sufficient to raise NAD^+^ levels in *rpd3Δ* cells to any significant degree. When paired however, the two adjustments were able to restore NAD^+^ levels in *rpd3Δ* back to those observed in WT cells. Interestingly, the NAD^+^ levels under these conditions were still slightly below that of WT cells with *BNA6-oe* and QA supplementation, suggesting that other factors downstream of *BNA6* may also be impacted because of *rpd3Δ*.

### Rpd3 binding to the *BNA2* promoter is altered by deleting Hst1 and vice versa

**Figure 5:**
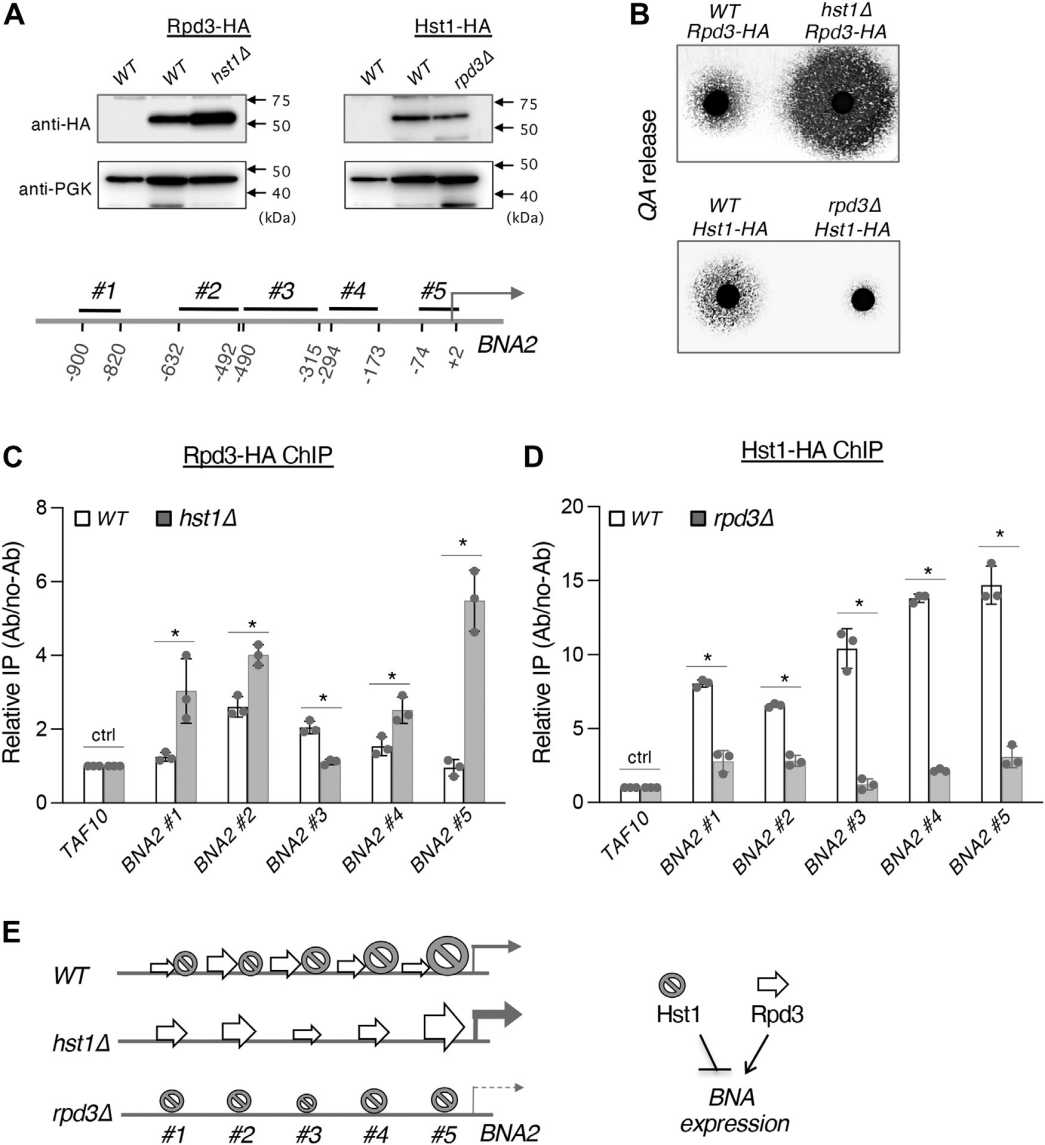
**Analysis of Rpd3 and Hst1 binding to the *BNA2* promoter.***A*, design of the chromatin immunoprecipitation (ChIP) studies. HA-tagged Hst1 was generated in both WT and *rpd3Δ* cells. HA-tagged Rpd3 was generated in both WT and *hst1Δ* cells. Expression was confirmed by Western blot analysis (*top*). BNA2 promoter regions for ChIP studies are shown as *BNA2 #1*, *#2*, *#3*, *#4*, and *#5* (*bottom*). *B*, confirming the QA cross-feeding phenotypes of HA-tagged strains. Both Rpd3-HA WT and Hst1-HA WT cells show WT levels of QA release. Deletion of *HST1* in Rpd3-HA WT cells increases levels of QA release. Deletion of *RPD3* in Hst1-HA WT cells decreases levels of QA release. *C*, ChIP analysis of Rpd3 binding to the *BNA2* promoter. The pattern of Rpd3 binding is altered in *hst1Δ* cells. Binding activity of Rpd3 is most significant near *BNA2 #2* in WT cells, which shifts to *BNA2 #5* in *hst1Δ* cells. Relative IP levels were normalized to *TAF10*. *D*, Hst1-binding activity is the highest near the transcription start site (*BNA2 #5*) in an ascending pattern. Hst1-binding activity is decreased when Rpd3 is absent. For *C* and *D*, the graphs are representative of the trend observed across three independent experiments. Error bars represent data from three technical replicates for each strain in an experiment. The *p* values are calculated using Student’s *t* test (∗*p* < 0.05; *ns*, not significant). *E*, model of Rpd3 and Hst1 binding to the *BNA2* promoter. BNA, biosynthesis of nicotinic acid; QA, quinolinic acid.

Next, we sought to investigate the interaction between Rpd3 and Hst1 at the promoter of the *BNA* genes. Hst1 has been shown to bind to the promoter of *BNA2* (38), which mediates the first and rate-limiting step of *de novo* QA synthesis. Therefore, we determined whether Rpd3 affects the binding activity of Hst1 at the *BNA2* promoter and vice versa. To achieve this, we carried out chromatin immunoprecipitation (ChIP) studies of various *BNA2* promoter fragment using HA-tagged Rpd3 (Rpd3-HA) and Hst1 (Hst1-HA) strains. Figure 5, *A* (*top*) shows Rpd3 and Hst1 protein levels used for this study. It appeared that deletion of *HST1* resulted in slightly increased expression of Rpd3-HA, whereas deletion of *RPD3* resulted in somewhat decreased levels of Hst1-HA (Fig. 5*A*, *top*). However, we did not expect this to have a significant influence on Rpd3 binding to the *BNA2* promoter. As shown in Figure 5, *A* (*top*), the Rpd3-HA WT strain showed a normal level of QA release and was still sensitive to the dosage of Hst1, as deleting *HST1* in this strain was able to significantly increase QA release (Fig. 5*B*, *top*). Similarly, deleting *RPD3* in Hst1-HA WT cells also drastically reduced QA release (Fig. 5*B*, *bottom*), suggesting that Hst1-HA remained an efficient repressor and was recruited to the *BNA2* promoter in both WT and *rpd3Δ* backgrounds. Figure 5, *A* (*bottom*) illustrates *BNA2* promoter fragments (*BNA2* #1, #2, #3, #4, and #5) used in ChIP studies. As shown in Figure 5, *C*, the binding activity of Rpd3 was increased near the middle of the *BNA2* promoter (*BNA2* #2 and #3) and tapered off toward the -1000 and 0 sites. Interestingly, when Hst1 is absent, Rpd3 appears to move away from the middle of the promoter (*BNA2* #3) and instead occupying the ends (*BNA2* #1, #2, #4, and #5) (Fig. 5*C*). In particular, we noted the highest increase of Rpd3 occupancy near site #5 (*BNA2* #5), directly proximal to the transcription start site. This is in accordance with expectation, as Hst1 was previously shown to exhibit the highest binding activity in this region of the *BNA2* promoter (38). Ultimately, when Hst1 is absent, Rpd3-binding distribution on the *BNA2* promoter is altered, with the most significant increase near the transcription start site. This result suggests that Hst1 may oppose Rpd3 binding at specific regions of the *BNA2* promoter. Next, we examined whether the absence of Rpd3 would affect Hst1 binding to the *BNA2* promoter. In agreement with previous studies (38), Hst1-binding activity was the highest near the transcription start site (*BNA2* #5) in a pattern ascending from the -1000 to 0 position (Fig. 5*D*). Interestingly, when Rpd3 is absent, not only was the ascending binding pattern of Hst1 abolished but also was the overall Hst1-binding activity significantly reduced. This result not only indicates that Rpd3 indeed affects Hst1-binding distribution at the *BNA2* promoter but also raises the possibility that Rpd3 may be important for Hst1 binding. However, the latter contradicts the expectation that Rpd3, as a positive regulator of *BNA* expression, would most likely oppose Hst1 binding. It is clear that Hst1 in the *rpd3Δ* background is still highly proficient in silencing *BNA* expression and limiting *de novo* activity (Figs. 4*A* and 5*B*) and remains bound at levels significantly above those at the *TAF10* control site (Fig. 5*D*). Otherwise, the *rpd3Δ*Hst1-*HA* cells would have shown a similar high QA release phenotype as seen in *hst1Δ* cells, which was not the case here (Fig. 5*B*). Therefore, it is more likely that the ascending binding pattern of Hst1 is due to higher Rpd3-binding activity toward the middle of the *BNA2* promoter and that in the absence of Rpd3, Hst1 is able to redistribute more evenly on the *BNA2* promoter. This conclusion supports a model in which Rpd3 may oppose Hst1 binding at specific regions of the *BNA2* promoter, possibly by preventing the overspreading of silenced chromatin, which is important for proper *BNA* gene expression. The cause of this generally reduced Hst1 binding may also be the reduced NAD^+^ levels exhibited by the *rpd3Δ* mutant (Fig. 2*E*). As an NAD^+^-dependent HDAC, the activity of Hst1 is expected to decrease under conditions of NAD^+^ limitation, which is reflected in the observed reduction in Hst1-binding activity. Supporting this, it has been reported that of all the sirtuins, Hst1 is most sensitive to NAD^+^ levels, responding quickly to perturbations to the NAD^+^ pool within the range of physiological concentration (37). It is also possible that without the opposing effect of Rpd3, Hst1 does not need to access the *BNA2* promoter frequently in order to maintain silent chromatin.

### Rpd3 and Hst1 deacetylate the N-terminal lysine residues of the core histone protein H4

**Figure 6:**
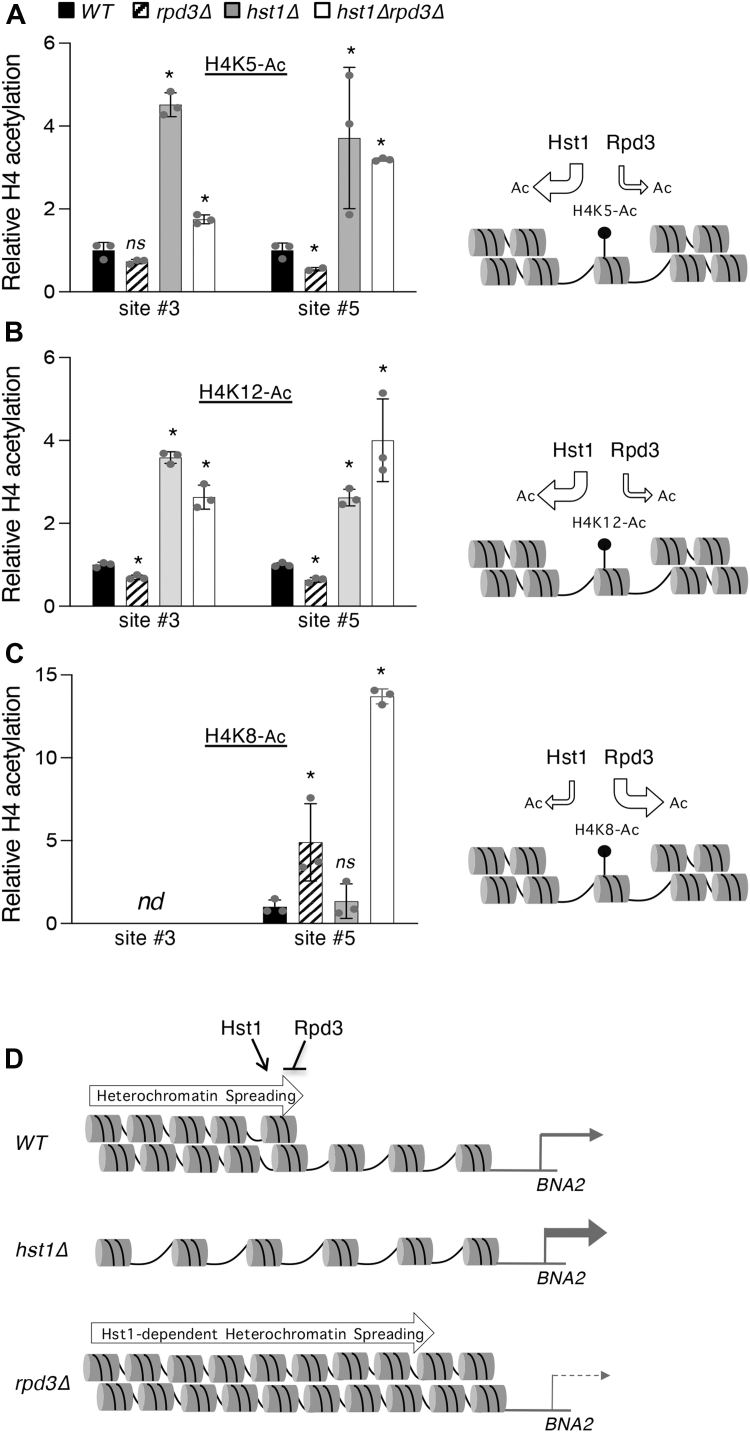
**Rpd3 and Hst1 have opposing effects on histone H4 acetylation status at the *BNA2* promoter.***A*, relative abundance of acetylated H4K5 (H4K5-Ac) at sites 3 and 5, depicted in Figure 5, *A*, of the *BNA2* promoter (*left*). Deletion of RPD3 slightly decreases the amount of H4K5-Ac, whereas deletion of *HST1* as well as deletions of *RPD3* and *HST1* together increase the level of H4K5-Ac, suggesting that Hst1 is the main deacetylase for this residue (*right*). *B*, relative abundance of H4K12-Ac at sites #3 and #5 of the *BNA2* promoter (*left*). *rpd3Δ* cells show reduced acetylation of H4K12, whereas *hst1Δ* and *hst1Δrpd3Δ* cells show increased acetylation of H4K12, suggesting that Hst1 is primarily responsible for the deacetylation of this residue (*right*). *C*, relative abundance of H4K8-Ac at sites #3 and #5 of the *BNA2* promoter (*left*). *rpd3Δ* and *hst1rpd3Δ* cells show increased acetylation of H4K8, whereas deletion of *HST1* alone does not have a significant influence on H4K8-Ac levels, suggesting that Rpd3 is the main deacetylase for this residue. Values are relative to levels of H4 protein-bound DNA in each strain, and all values are normalized to those of WT cells. The graphs are representative of the trend observed across three independent experiments. Error bars represent data from three technical replicates for each strain in an experiment. The *p* values are calculated using Student’s *t* test (∗*p* < 0.05; *nd*, not detected; *ns*, not significant). *D*, model of *BNA2* expression and putative chromatin structure produced by the effects of Rpd3 and Hst1. BNA, biosynthesis of nicotinic acid.

Having determined how Rpd3 and Hst1 interact in the binding of the *BNA2* promoter, we then sought to determine how each HDAC affects the histone acetylation status of the *BNA2* promoter. In investigating this question, we examined histone acetylation at two of the promoter sites indicated in Figure 5, *A* in order to account for any possible local effects of each HDAC. Rpd3 binding peaks at the middle of the promoter near site #3 (Fig. 5*C*), whereas Hst1 binding peaks immediately upstream of the transcription start site at site #5 (Fig. 5*D*). Rpd3 has previously been shown to deacetylate a wide variety of lysine residues on the N-terminal tail of core histone proteins, among these being H4K5, H4K8, H4K12, H3K9, H3K14, H3K18, H3K23, H3K27, and H2AK7 (54, 55, 56). Hst1, on the other hand, has been identified as a deacetylase of H3K4 (57), H4K5, and potentially H4K12 (58). Owing to Rpd3 and Hst1 having H4K5 and H4K12 as shared targets, we chose to focus on histone H4 as a potential site of competition between Rpd3 and Hst1. Our ChIP results showed that Hst1 is the primary deacetylase for H4K5-Ac and H4K12-Ac at the *BNA2* promoter, as the levels of H4K5-Ac and H4K12-Ac were increased in *hst1Δ* cells (at both site #3 and site #5). Deleting *RPD3* slightly yet significantly decreases the level of H4K5-Ac and H4K12-Ac (at site #5, near the transcription start site) (Fig. 6, *A* and *B*). This is interesting in light of the fact that deletion of *RPD3* was previously associated with increased acetylation of both these residues (56). In this case, however, Rpd3 seems to interfere with the deacetylation of both by an unknown mechanism, whereas Hst1 appears to be the primary deacetylase for these residues at the *BNA2* promoter. These results suggest that in the absence of Rpd3, another HDAC, likely Hst1, deacetylates H4K5-Ac and H4K12-Ac more efficiently at the *BNA2* promoter. Supporting this, deleting *HST1* in *rpd3Δ* cells restored acetylation to similar levels observed in the *hst1Δ* cells (Fig. 6, *A* and *B*).

Interestingly, the acetylation patterns seen for H4K8 differed from that of H4K5 and H4K12 (Fig. 6*C*). H4K8-Ac was comparatively difficult to detect at site #3, possibly indicating that it is less abundant than the other acetyl marks. Moreover, H4K8-Ac abundance was increased in *rpd3Δ* cells, in conformity with the previously established role of Rpd3 as a deacetylase of H4K8. On the other hand, any enrichment of H4K8-Ac in *hst1Δ* cells was minor and nonsignificant, unlike acetylation of the other two residues. However, H4K8-Ac levels were strongly increased in *hst1Δrpd3Δ* cells, pointing to the possibility of synergy between the two HDACs in the deacetylation of H4K8. These results suggest that Hst1 is the primary deacetylase of H4K5 and K4K12 at the *BNA2* promoter, whereas Rpd3 works to limit deacetylation of these residues in this context, in close agreement with the antagonism seen between the two in the regulation of *BNA* expression and *de novo* NAD^+^ metabolism. On the other hand, the two HDACs also appear to both deacetylate H4K8 to some degree, suggesting a variety of different interactions between Rpd3 and Hst1 at the *BNA2* promoter, all of which ultimately combine to regulate *BNA2* expression in a specific manner. Ultimately, Hst1 seems to promote repressed chromatin status at the *BNA2* promoter (Fig. 6*D*). H4 acetylation (Figure 6, *A–C*), Rpd3 binding (Fig. 5*C*), and *BNA2* expression are all increased in *hst1Δ* cells (Fig. 4*A*). On the other hand, Rpd3 activity is likely associated with derepressed chromatin status in this context (Fig. 6*D*); H4K5 and H4K12 acetylation is somewhat reduced in *rpd3Δ* cells (Fig. 6, *A* and *B*), whereas *BNA2* expression is very strongly decreased (Fig. 4*A*). Moreover, the reduced binding of Hst1 to the *BNA2* promoter in *rpd3Δ* cells (Fig. 5*D*) may in part be caused by this putative repressed chromatin structure.

### *De novo* NAD^+^ metabolism, NA–NAM salvage, NR salvage, and transport of NAD^+^ precursors are integrated by Rpd3 and Hst1

**Figure 7:**
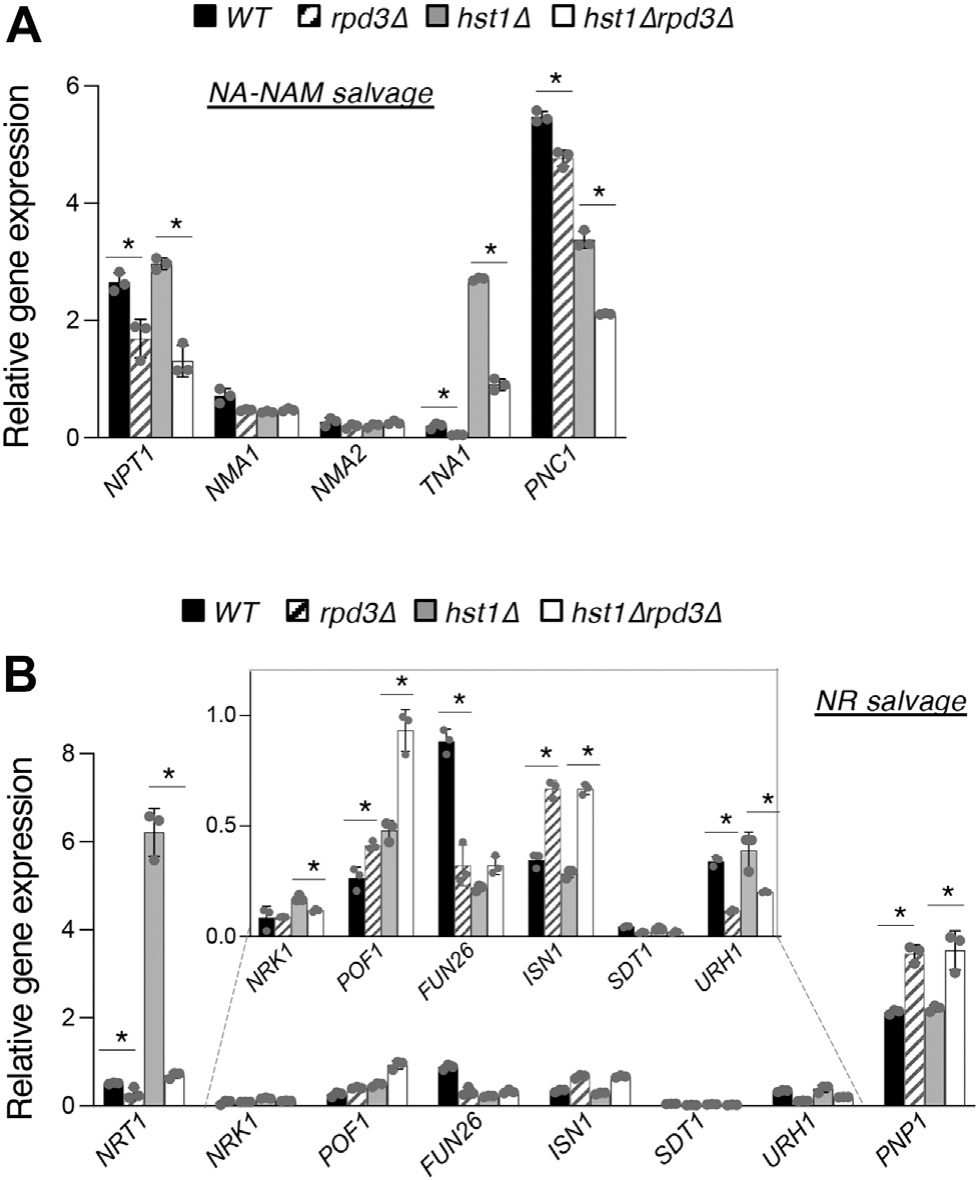
**Rpd3 and Hst1 regulate different downstream target genes in NA–NAM and NR salvage pathways.***A*, relative expression analysis of the genes of the NA–NAM (*left*) and NR (*right*) salvage pathways in WT, *rpd3Δ*, *hst1Δ*, and *hst1Δrpd3Δ* cells by quantitative PCR (qPCR). *B*, relative expression analysis of the genes of the NR salvage pathway in WT, *rpd3Δ*, *hst1Δ*, and *hst1Δrpd3Δ* cells by qPCR. All values shown are relative expression levels normalized to *TAF10* as a control. The graphs are representative of the trend observed across three independent experiments. Error bars represent data from three technical replicates for each strain in an experiment. The *p* values are calculated using Student’s *t* test (∗*p* < 0.05; *ns*, not significant). NA, nicotinic acid; NAM, nicotinamide; NR, nicotinamide riboside.

Next, we further examined how Rpd3 and Hst1 may affect other branches of NAD^+^ metabolism. We tested the expression of genes known to be involved in NA–NAM and NR salvage in WT, *rpd3Δ*, *hst1Δ*, and *hst1Δrpd3Δ* cells by quantitative RT–PCR (qRT–PCR) (Fig. 7*A*). As shown in Figure 7, *A*, differential expression of NA–NAM salvage genes including *NPT1*, *TNA1*, and *PNC1* was observed. A significant decrease in *NPT1* expression was seen in *rpd3Δ* and *hst1Δrpd3Δ* cells (Fig. 7*A*). These changes may account for the low NAD^+^ phenotype of *rpd3Δ* and *hst1Δrpd3Δ* cells in SC media (Figs. 2*E* and 4*F*) since Npt1 is a major NAD^+^ biosynthesis enzyme in NA–NAM salvage (Fig. 1*A*). Decreased expression of *PNC1* in *rpd3Δ* and *hst1Δ* cells with a further decrease in *hst1Δrpd3Δ* cells suggests that the two HDACs positively and independently regulate *PNC1*. The significant reduction of *PNC1* expression in *hst1Δrpd3Δ* cells correlates with the high levels of NA–NAM accumulation seen in these cells (Fig. 2*B*). Interestingly, *rpd3Δ* cells showed a contrasting expression pattern of *URH1* and *PNP1* (Fig. 7*B*), both being nucleosidases that convert NR to NAM (Fig. 1*A*). Since Urh1 has been suggested to be the primary mediator of this reaction (39), decreased *URH1* expression likely contributes to the increased NR (Fig. 2*D*) and reduced NA–NAM (Fig. 2*B*) observed in *rpd3Δ* cells. Moreover, increased expression of *ISN1* (Fig. 7*B*), a nucleotidase converting NMN to NR, may contribute to increased NR observed in *rpd3Δ* cells (Fig. 2*D*). Interestingly, we observed increased expression of *POF1* in *rpd3Δ* and *hst1Δ* cells with a further increase in *hst1Δrpd3Δ* cells. Although Pof1 has been shown to convert NMN to NAD^+^ (35), its cellular function is not fully understood. In addition, we noted that the two HDACs appear to regulate the QA–NA transporter *TNA1* and the NR transporter *NRT1* in a manner closely resembling the *BNA* genes, with decreased expression observed in *rpd3Δ* cells and increased expression in *hst1Δ* cells (Fig. 7*A*). In both cases, the *hst1Δrpd3Δ* cells showed a clearly intermediate level of expression most closely matching that of WT. It should be noted, however, that this altered *TNA1* expression is likely not a significant contributor to the *de novo* pathway defects observed in *rpd3Δ* and *hst1Δ* cells, with both strains exhibiting phenotypes opposite those expected in association with aberrant Tna1 activity. We also found that *rpd3Δ*, *hst1Δ*, and *hst1Δrpd3Δ* cells evinced markedly reduced levels of *FUN26* expression, a vacuolar NR transporter (41). Overall, these gene expression results are in line with observed aberrant NAD^+^ precursor phenotypes. However, it remains unclear what accounts for the significant increase of NR production seen in *hst1Δrpd3Δ* cells (Fig. 2*D*).

## Discussion

In this study, we have established a role for Rpd3 as a positive regulator of *de novo* NAD^+^ metabolism. In particular, it opposes the negative regulation of the *BNA* genes by another HDAC Hst1 (Figs. 4*A* and 6). The *rpd3Δ* and *hst1Δ* mutants show contrasting QA release phenotypes. Interestingly, *hst1Δ* appears to override *rpd3Δ*, and the *hst1Δrpd3Δ* double mutant behaves like the *hst1Δ* single mutant (Fig. 1, *C* and *D*), with the exception of intracellular QA levels (Fig. 1*E*). Our studies suggest that the high *BNA6* expression in *hst1Δ* and *hst1Δrpd3Δ* cells (Fig. 4*A*) may facilitate QA assimilation and therefore reduce intracellular QA accumulation. The same pattern of reduced *de novo* metabolism in *rpd3Δ* cells and increased metabolism in *hst1Δ* cells is seen for several intermediates upstream of QA, namely 3-HK (Fig. 3*C*) and 3-HA (Fig. 3*D*). Moreover, *rpd3Δ* cells accumulate TRP (Fig. 3*A*), likely because of reduced Bna2 activity at the first and rate-limiting step of *de novo* NAD^+^ metabolism, whereas *hst1Δ* and *hst1Δrpd3Δ* cells accumulate less TRP than WT cells, reflecting increased consumption *via de novo* metabolism. Rpd3 has also been implicated in recycling of Tat2, a TRP transporter. The growth of a strain defective in Tat2-depedent transport is significantly inhibited under low TRP conditions by deletion of *RPD3* (59). We saw, however, that *rpd3Δ* cells remain proficient for TRP uptake, accumulating the metabolite above levels seen in WT cells (Fig. 3*A*). This suggests that reduced assimilation of TRP into NAD^+^ *via* the *de novo* pathway may contribute to the growth defects of *rpd3Δ* cells in low TRP media (59). In addition to defective *de novo* NAD^+^ metabolism, cells lacking Rpd3 also show altered NA–NAM and NR salvage activities. Although contrasting NA–NAM release phenotypes are also observed in *rpd3Δ* and *hst1Δ* mutants (Fig. 2, *A* and *B*), it appears that different downstream genes are dysregulated in each mutant. The lower NA–NAM levels in the *rpd3Δ* mutant (Fig. 2, *A* and *B*) are likely because of overall lower NAD^+^ levels (Fig. 2, *E* and *F*). It is also possible that reduced *URH1* expression (Fig. 7*B*) results in less NR to NAM conversion (24). In *hst1Δ* (including *hst1Δrpd3*Δ) cells, increased intracellular NA–NAM levels (Fig. 2*B*) are likely because of increased expression of the NA transporter *TNA1* as well as reduced nicotinamidase *PNC1* expression (Fig. 7*A*). On the other hand, the *rpd3Δ* and *hst1Δ* mutants also show contrasting NR release patterns, but this time, the *rpd3Δ* mutant releases more NR (Fig. 2*C*). The *hst1Δ* mutant appears to release less NR because most NR is accumulated intracellularly (Fig. 2*D*) because of high expression of the NR transporter *NRT1* (Fig. 7*B*). Increased NR release in the *rpd3Δ* mutant is likely because of decreased *NRT1* expression (Fig. 7*B*). Notably, *rpd3Δ* and *hst1Δ* have a synergistic effect on NR production (Fig. 2*D*), which has also been seen for the expression of other genes involved in NR salvage including *POF1* (Fig. 7*B*). Overall, our results show that Rpd3 and Hst1 opposingly coregulate the *de novo BNA* genes, yet they seem to affect different target genes in the salvage pathways. Among the genes examined, only the NAD^+^ precursor transporters *NRT1* and *TNA1* (Fig.7, *A* and *B*) appear to be regulated by the two HDACs in a manner resembling the *BNA* genes (Fig. 4*A*).

Our studies also indicate that observed NAD^+^ deficiencies in *rpd3Δ* cells are likely caused by various factors discussed previously depending on specific growth conditions. In standard NA-rich growth media, *de novo* pathway is repressed by the NAD^+^-dependent Hst1. Therefore, observed low NAD^+^ defects in *rpd3Δ* (and *hst1Δrpd3Δ*) cells (Fig. 2*E*) likely result from increased NR production in conjunction with a blockage in NR flow to NA–NAM salvage and reduced NA assimilation because of decreased expression of *NPT1* (Fig. 7*A*) and *URH1* (Fig. 7*B*). The effects of *rpd3Δ* and *hst1Δ* on *de novo* pathway activity are better understood since this pathway can be studied in defined NA-free growth media (Fig. 2*F*). Under this condition, observed NAD^+^ deficiencies are mainly because of reduced *BNA* expression, and so deleting *hst1Δ* overrides *rpd3Δ* and increases NAD^+^ levels (Fig. 2*F*). We also show that dysregulation of multiple *BNA* genes contributes to NAD^+^ deficiencies in *rpd3Δ* cells, since overexpressing individual rate-limiting *BNA2* gene and the *BNA6* gene was not sufficient to restore NAD^+^ levels (Fig. 4*E*). However, we were able to restore the levels of NAD^+^ in *rpd3Δ* cells by supplementing QA and overexpressing *BNA6* (Fig. 4*F*). These studies confirm that Rpd3 is important for optimal *BNA* gene expression and *de novo* NAD^+^ synthesis. To understand the mechanisms of Rpd3- and Hst1-mediated regulation of the *BNA* expression, we carried out ChIP studies to study the binding distributions of Rpd3 and Hst1 on the promoter of the *BNA2* genes (Fig. 5, *C* and *D*). The results gathered point toward a model in which Hst1 serves to limit the distribution pattern of Rpd3 on the *BNA2* promoter. Rpd3 also appears to affect the interaction of Hst1 with the *BNA2* promoter, but the effect is less significant, as the overall Hst1-binding activity at the *BNA2* promoter is reduced in *rpd3Δ* cells (Fig. 5, *C*–*E*). It is possible that Hst1 activity is reduced in *rpd3Δ* cells because of reduced NAD^+^ levels. This is interesting in light of the fact that Hst1 appears to work downstream of Rpd3, with *hst1Δ* sufficient to raise *BNA* expression and QA production to a similar extent in WT and *rpd3Δ* backgrounds. Therefore, we anticipate that additional factors may also play a role in the antagonistic *BNA* gene expression regulation exerted by Rpd3 and Hst1. One possibility is that specific transcription factors and/or chromatin remodeling factors are recruited to or excluded from the *BNA* promoter by specific chromatin modifications effected by the two HDACs.

Similar instances of antagonism between Rpd3 and other sirtuins have been noted in previous studies, with Rpd3 opposing repression of the silent mating-type loci by Sir2 and thereby promoting transcription (55). Rpd3 has also been shown to limit Sir2 spreading at telomere boundaries (60). While it is typical for HDACs to serve as chromatin silencers (61), Rpd3 has long been noted for seeming to promote transcription of certain genes (62). Interestingly, it was previously noted that the role played by Rpd3 in opposing Sir2 activity was indeed mediated by its catalytic HDAC activity, but that the effect did not depend on any of the known histone targets of Rpd3 (55). Indeed, there are many examples of HDACs acting on nonhistone proteins (63, 64), making the possible avenues of HDAC-dependent regulation quite extensive. Moreover, restoration of silencing by *RPD3* deletion in a *sir2*-catalytic-dead strain was dependent on Hst3 activity (55), highlighting the complementarity and interactive roles of different HDACs. In the case of *de novo* pathway regulation however, our QA cross-feeding screen (Fig. 1*B*) did not indicate the involvement of any other HDACs aside from Rpd3 and Hst1. It appears that some or all *BNA* genes may be regulated in a unique fashion. For example, Sir2 has previously been identified as a negative regulator of *BNA1* expression, with *sir2Δ* cells showing *BNA1* expression increased by approximately twofold (52). The positive regulator Bas1–Pho2, for instance, appears to affect each *BNA* gene to significantly varying degrees (25). We have previously seen as well that *BNA7* is insensitive to many of the factors that regulate other *BNA* genes (38). It does appear that Rpd3 and Hst1 are ubiquitous regulators of *de novo* NAD^+^ metabolism; however, this property may not extend to other regulators of the pathway. It will likely be necessary to study each *BNA* gene individually in order to fully elaborate the intricacies of *de novo* pathway regulation.

It has also been shown that treatment with sodium butyrate and other HDAC inhibitors is able to suppress aberrant *de novo* metabolism in Huntingtin-expressing cells in a similar fashion to *ume1Δ* and *rxt3Δ* (51), whereas treatment with the sirtuin inhibitor NAM produces effects on *BNA* expression similar to those observed in *hst1Δ* (38). These studies suggest that the catalytic activities of each HDAC are required at least to some extent and that the role each plays is not purely structural. In agreement with this notion, both HDACs seemed to have a rather significant effect on the acetylation status of histone H4. In particular, we found that the abundance of H4K5-Ac (Fig. 6*A*) and H4K12-Ac (Fig. 6*B*) was significantly increased in *hst1Δ* and *hst1Δrpd3Δ* cells, whereas H4K8-Ac abundance was significantly increased in *rpd3Δ* cells and very greatly raised in *hst1Δrpd3Δ* cells (Fig. 6*C*). Altogether, these results seem to implicate Hst1 as the major deacetylase active at the *BNA2* promoter and, together with increased *de novo* activity and *BNA* expression observed in *hst1Δ* cells, point toward a role for Hst1 in establishing a repressive chromatin architecture at the *BNA* promoters. Conversely, Rpd3, with the exception of H4K8-Ac, appears to generally limit deacetylation at the *BNA2* promoter. In combination with decreased *BNA* expression seen in *rpd3Δ* cells, Rpd3 appears to act in the capacity of a positive regulator by limiting Hst1-dependent deacetylation.

Though the specific mechanism of this antagonism between Rpd3 and Hst1 remains to be further investigated, there are several possibilities suggested by these results. Limitation of Hst1-dependent deacetylation by Rpd3 may proceed by means of competition for the same targets, such as H4K5-Ac and H4K12-Ac, wherein limited deacetylation by Rpd3 prevents recognition and full deacetylation by Hst1. It has previously been reported that certain HDACs may be recruited by a specific acetyl mark. For instance, H4K16-Ac, a target of Sir2, has been shown to promote the binding of Sir2 to chromatin (65). It is also possible that other acetyl marks may be targeted primarily by Rpd3, potentially including H4K8-Ac, reconfiguring the chromatin so as to interfere with Hst1 activity at different marks, the net result of which might be decreased silencing by Hst1. We saw that deletion of *RPD3* unexpectedly decreased Hst1 binding to the *BNA2* promoter (Fig. 5*D*); one possible explanation for this is that the repressive heterochromatin structure formed by Hst1 HDAC activity in the absence of Rpd3 might then limit the continued binding of Hst1 itself, with only enough Hst1 binding to maintain the heterochromatin structure. Finally, histone modifications made by Rpd3 may also be involved in the recruitment of additional chromatin remodeling factors or transcription factors to the *BNA2* promoter, which may then interact with Hst1. It has previously been established that the copper-sensing transcription factor Mac1 (38) and the Bas1–Pho2 complex (25) regulate *BNA* expression. How these factors, and possibly others, might interact with Rpd3 and Hst1 is a matter for future study.

It appears ultimately that Hst1 is involved in the formation of heterochromatin at the *BNA2* promoter *via* deacetylation of the N-terminal lysines of H4, whereas Rpd3 opposes Hst1 HDAC activity and maintains a less repressed chromatin structure (Fig. 6*D*). Whether Rpd3 and Hst1 target additional residues on other histones remains a topic for further study. Rpd3 has been shown to target a very wide group of acetyl-lysine residues across several different histone proteins, including H3K9, H3K14, H3K18, H3K23, H3K27, and H2AK7 (54), whereas Hst1 is known to deacetylate H3K4 (57) and may also deacetylate H4K16 in the absence of Sir2 and Pde2 (66). In addition, it remains to be investigated what effect each acetyl-lysine mark has on *BNA* expression and chromatin structure, and whether Rpd3 and Hst1 do indeed regulate heterochromatin formation in the manner proposed (38, 55).

In summary, Rpd3 and Hst1 appear to exert opposing influence on *de novo* NAD^+^ metabolism and also integrate the regulation of several disparate branches of NAD^+^ metabolism. This set of regulators helps coordinate a variety of inter-related metabolic signals in budding yeast. Further work will be required to explore the detailed mechanistic interactions among these regulators and to establish the means by which they compete and cooperate to influence the cellular pools of NAD^+^ and its precursors. Rpd3 and Hst1, having homologs in human HDAC1 and SIRT1, respectively, represent links between the study of the epigenetic regulation of NAD^+^ metabolism and various disease states. Aberrant NAD^+^ metabolism and associated dysregulation of sirtuin activities have been implicated in a number of human disorders (5, 6, 18, 19, 20, 21, 22). HDAC1 has also been associated with a variety of diseases, and HDAC inhibition generally has received attention as a therapeutic intervention in a variety of contexts (67, 68, 69). It is currently unclear whether sirtuins and HDAC1 also regulate NAD^+^ metabolism in other organisms by similar mechanisms. It would be interesting to determine what other genes are also coregulated by Hst1 and Rpd3 in future studies. Notably, many NAD^+^ intermediates also have a manifold set of relationships with cellular health. For instance, the intermediates of the *de novo* pathway have diverse interactions with infectious mechanisms, metabolic stress, and immune signaling (45). Altogether, this work contributes to the elaboration of the relations by which NAD^+^ metabolism is governed and helps to connect different branches of NAD^+^ metabolism among each other.

## Experimental procedures

### Yeast strains, growth media, and plasmids

Yeast strain BY4742 *MATα his3Δ1 leu2Δ0 lys2Δ0 ura3Δ0* acquired from Open Biosystems (70) was used as the parental WT strain for this study. Standard growth media including synthetic defined (SD) minimal, SC, and yeast extract/peptone/dextrose–rich media were made as described (71). Special NA-free SD and NA-free SC were made by using niacin-free yeast nitrogen base acquired from Sunrise Science Products. Gene deletions were carried out by replacing the coding regions of WT genes with gene-specific PCR products generated using either the p*AG32-hphMX4* (72) or the reusable *loxP*-*kanMX-loxP* (pUG6) (73) cassettes as templates. Multiple gene deletions employed a galactose-inducible Cre recombinase to remove the *loxP*-*kanMX-loxP* cassette, followed by another round of gene deletion (73). The HA epitope tag was added to target genes directly in the genome using the *pFA6a-3HA-kanMX6* (for *HST1*) or p*FA6a-kanMX6-PGAL1-3HA* (for *RPD3*) plasmids as template for PCR-mediated tagging (74). The *BNA6-oe* and *BNA2-oe* plasmids p*ADH1*-*BNA6* (75) and p*ADH1-BNA2-URA3* were made in the integrative p*PP81* (*LEU2*) and p*PP35* vectors, respectively. After Pac1 digestion, the linearized plasmids were introduced to yeast cells as described (71). The p*ADH1*-*BNA2-URA3* plasmid was made by cloning the Not1–Nhe1 DNA fragment from the p*ADH1*-*BNA2* plasmid (38) into p*PP35* cut with Not1 and Nhe1.

### QA, NR, and NA–NAM cross-feeding plate assays

These assays employed specific mutants, which depend on QA, NR, or NA–NAM for growth, as “recipient cells” and yeast strains of interest as “feeder cells.” First, recipient cells were plated as a lawn on a solid agar plate (∼10^4^ cells/cm^2^). Next, ∼2 × 10^4^ cells of each feeder cell strain (2 μl cell suspension made in sterile water at an absorbance of 1 at 600 nm) were spotted onto the lawn of recipient cells. Plates were then incubated at 30 °C for 3 days. Since the growth media do not contain the NAD^+^ intermediates needed for the growth of recipient cells, the extent of the recipient cell growth indicates the levels of specific NAD^+^ intermediates released by feeder cells. QA crossfeeding was carried out on SC or SD using the QA-dependent *npt1Δnrk1Δbna4Δ* mutant. NR crossfeeding was carried out on yeast extract/peptone/dextrose or SC using the NR-dependent *npt1Δbna6Δpho5Δ* mutant. NA–NAM crossfeeding was carried out on NA-free SC or NA-free SD using the NA–NAM-dependent *bna6Δnrk1Δnrt1Δ* mutant.

### Measurement(s) of NAD^+^, NADH, QA, NR, and NA–NAM

Total intracellular levels of NAD^+^ and NADH were determined using enzymatic cycling reactions as described (76). In brief, approximately one absorbance at 600 nm unit (one absorbance at 600 nm unit = 1 × 10^7^ cells/ml) cells grown to early logarithmic phase in SC (∼6 h growth from absorbance of 0.1 at 600 nm) was collected in duplicate by centrifugation. Acid extraction was performed in one tube to obtain NAD^+^, and alkali extraction was performed in the other to obtain NADH for 40 min at 60 °C. Amplification of NAD^+^ or NADH in the form of malate was carried out using 3 μl or 4 μl of neutralized acid– or alkali-extracted lysate in 100 μl of cycling reaction for 1 h at room temperature. The reaction was terminated by heating at 100 °C for 5 min. Next, malate produced from the cycling reaction was converted to oxaloacetate and then to aspartate and a-ketoglutarate by the addition of 1 ml malate indicator reagent for 20 min at room temperature. The reaction produced a corresponding amount of NADH as readout, which was measured fluorometrically with excitation at 365 nm and emission monitored at 460 nm. Standard curves for determining NAD^+^ and NADH concentrations were obtained as follows: NAD^+^ and NADH were added into the acid and alkali buffer to a final concentration of 0, 2.5, and 7.5 μM, which were then treated with the same procedure along with other samples. The fluorometer was calibrated each time before use with 0, 5, 10, 20, 30, and 40 μM NADH to ensure that the detection was within a linear range. Levels of NAD^+^ intermediates (QA, NR, and NA–NAM) were determined by a liquid-based cross-feeding bioassay as previously described (39, 41, 75) with modifications. To prepare cell extracts for intracellular NAD^+^ intermediate determination, approximately 200 absorbance at 600 nm unit (for NR and NA–NAM) or 900 absorbance at 600 nm unit (for QA) donor cells grown to late-logarithmic phase in SC (∼16 h growth from an absorbance of 0.1 at 600 nm) were collected by centrifugation and lysed by bead-beating (Biospec Products) in 400 μl (per 200 absorbance at 600 nm unit cells) ice-cold 50 mM ammonium acetate solution. The supernatant was collected by centrifugation, and the pellet was extracted two more times with 600 μl ice-cold 50 mM ammonium acetate solution, which generates 1600 μl cell lysate. After filter sterilization, 100 to 200 μl of clear extract (2.5 ml for QA) was used to supplement 8 ml cultures of recipient cells with starting absorbance of 0.05 at 600 nm in SC. To determine extracellular NAD^+^ intermediate levels, 20 ml supernatant of donor cell culture was collected, filter-sterilized, and then 4 ml was added to recipient cell culture in 2× SC to a final volume of 8 ml with total starting absorbance of 0.05 at 600 nm. A control culture of recipient cells in SC without supplementation was included in all experiments. For measuring relative QA levels, *npt1Δnrk1Δbna4Δ* and *npt1Δnrk1Δbna1Δ* mutants were used as recipient cells. The *npt1Δbna6Δpho5Δ* recipient cells were used to measure relative NR levels. To measure relative NA–NAM levels, the *bna6Δnrk1Δnrt1Δ* recipient cells were grown in NA-free SC. After incubation at 30 °C for 24 h, growth of the recipient cells (absorbance at 600 nm) was measured and normalized to the cell number of each donor strain. Absorbance at 600 nm readings were then converted to concentrations of QA, NR, and NA–NAM using the standard curves established as previously described (39, 41).

### MS analysis of metabolite levels

Metabolomic data were acquired at the UC Davis West Coast Metabolomics Center. For each sample, approximately 300 absorbance at 600 nm unit (for QA) cells grown to late-logarithmic phase in SC (∼16 h growth from an absorbance of 0.1 at 600 nm) were collected by centrifugation. Snap freezing was achieved by dry ice. Frozen cell pellets were kept in Eppendorf tubes and then subject to metabolite extraction and MS analysis. Cells were extracted following recommendations published before (77). GC-TOF was performed with an Agilent 6890 gas chromatography instrument with an Rtx-5Sil MS column coupled to a Leco Pegasus IV time of flight mass spectrometer (78). For data processing, ChromaTOF version 4.50.8 was used in conjunction with the BinBase algorithm as previously described (79). Metabolite identifications were performed according to the Metabolomics Standards Initiative by using chromatography-specific databases in conjunction with Mass Bank of North America (http://massbank.us) and NIST 20 mass spectral libraries (80).

### qPCR analysis of gene expression levels

Approximately 40 absorbance at 600 nm unit cells grown to early logarithmic phase in SD (6 h growth from an absorbance of 0.1 at 600 nm) were collected by centrifugation. Total RNA was isolated using GeneJET RNA purification Kit (Thermo Fisher Scientific), and complementary DNA was synthesized using QuantiTect Reverse Transcription kit (Qiagen) according to the manufacturer’s instructions. For each qPCR, 50 ng of complementary DNA and 500 nM of each primer were used. qPCR was run on Roche LightCycler 480 using LightCycler 480 SYBR green I Master Mix (Roche) as previously described (35). Average size of the amplicon for each gene was ∼150 bp. The target mRNA transcript levels were normalized to TAF10 transcript levels.

### Protein extraction and Western blot analysis

Approximately 50 absorbance at 600 nm unit cells grown in SC to early logarithmic phase (absorbance of ∼1 at 600 nm) were collected by centrifugation. The cell lysate was obtained by bead beating in lysis buffer: 50 mM Tris–HCl, pH 7.5, 100 mM NaCl, 1% Triton X-100, 5 mM EDTA (pH = 8), 1 mM PMSF, and protease inhibitor cocktail (Pierce). The protein concentration was measured using the Bradford assay (Bio-Rad), and 20 to 25 μg (Hst1, Bna, and PGK) or 40 μg (Rpd3) of total protein was loaded in each lane. After electrophoresis, the protein was transferred to a polyvinylidene fluoride membrane (GE Healthcare). Blocking was carried out using OneBlock Western-CL Blocking buffer. The membranes were then washed, blotted with either anti-HA rabbit antibody (Cell Signaling; catalog no.: 3724S) or anti-PGK mouse antibody (Invitrogen; catalog no.: 459250). Protein was visualized using antimouse or anti-rabbit immunoglobulin antibody conjugate to horseradish peroxidase (Invitrogen) and the ECL reagents (Amersham, GE). The chemiluminescent image was analyzed using the Amersham Imager 600 (GE) system and software provided by the manufacturer.

### ChIP assay

Approximately 500 absorbance at 600 nm unit cells grown to early logarithmic phase in SD were crosslinked with 1% formaldehyde for 30 min at room temperature and stopped by adding glycine to a final concentration of 125 mM. Cells were pelleted by centrifugation and washed two times with cold Tris-buffered saline (20 mM Tris–HCl, pH 7.5, 150 mM NaCl). Cells were lysed by bead beating in 1 ml of FA-140 lysis buffer (50 mM Hepes, 140 mM NaCl, 1% Triton X-100, 1 mM EDTA, 0.1% sodium deoxycholate, 0.1 mM PMSF, and 1× protease inhibitor cocktail [Pierce]) (81). The cell lysate was drawn off the beads and centrifuged at a maximum speed (13,200 rpm) for 30 min at 4 °C. The chromatin pellet was resuspended in 1 ml of FA-140 lysis buffer and sonicated on ice eight times with 20 s pulses using a Branson 450 Sonicator (output control set at 1.5 and duty cycle held at constant) to shear chromatin to an average length of ∼500 bp. Sonicated chromatin solution was centrifuged twice at 10,000 rpm for 10 min at 4 °C. The supernatant was then aliquoted into two tubes (labeled “IP” [immunoprecipitated] and “no-Ab” [no antibody]). The IP samples were incubated overnight at 4 °C with anti-HA monoclonal antibody (catalog no.: ab1424; Abcam) at a dilution of 1:150. Both IP and no-Ab samples were incubated with 60 μl of ChIP-grade protein G beads (Cell Signaling Technology) for 2 h at 4 °C and then washed as described (81). DNA was then eluted from the beads two times with 125 μl of elution buffer (5× TE, 1% SDS). The combined DNA solution and input samples were incubated at 65 °C overnight to reverse the crosslinking. The purified DNA samples were analyzed by qPCR. The amount of immunoprecipitated specific promoter DNA was determined relative to no-Ab DNA. For quantitation of histone acetyl-lysine marks, each mark, along with the H4 protein itself, was precipitated as aforementioned using the following antibodies: ab10158, Abcam (H4); 71-290-6, Invitrogen (H4K5-Ac); ab15823, Abcam (H4K8-Ac); ab46983, and Abcam (H4K12-Ac). The amount of each mark was determined relative to no-Ab DNA and H4-bound DNA.

## Data availability

All data are contained within this article.

## Conflict of interest

The authors declare that they have no conflicts of interest with the contents of this article.
